# Editorial: thematic issue on thermophiles

**DOI:** 10.1093/femsec/fiag045

**Published:** 2026-04-29

**Authors:** Olga V Golyshina, Tillmann Lüders, Peter N Golyshin

**Affiliations:** School of Environmental and Natural Sciences, College of Science and Engineering, Bangor University, Deiniol Road, LL57 2DG Bangor, United Kingdom; Faculty of Biology, Chemistry and Earth Sciences, University of Bayreuth, 95440 Bayreuth, Germany; School of Environmental and Natural Sciences, College of Science and Engineering, Bangor University, Deiniol Road, LL57 2DG Bangor, United Kingdom

## Abstract

The most ancient pathway of carbon fixation, the acetyl-CoA pathway, is a time capsule that uncovers primordial traits in the evolution of life.

Thermophilic micro-organisms thrive at temperatures above 50°C and inhabit a wide range of high-temperature environments across our planet. Research on thermophiles is essential for deepening our understanding of their unique physiologies, phylogenomic diversity, geographic distribution, and the molecular mechanisms that enable their adaptation to extreme conditions. Such studies also shed light on the origins and evolution of life on Earth and pave the way for industrial applications of thermophiles, their enzymes, and metabolic products.

The 16^th^ International Congress on Thermophiles was held in late August and early September 2023 in Bangor, North Wales, UK, an area of outstanding natural beauty nestled between the Snowdonia Mountains and the Irish Sea coastline. This biennial conference series, inaugurated in 1990 in Viterbo, Italy, covers a broad spectrum of topics including ecology, evolution, phylogeny, genomics, metagenomics, molecular biology, physiology, astrobiology, and biotechnology. The congress provides a platform for researchers in the field of thermophilic studies to present their latest discoveries, exchange new concepts and methodologies, and foster scientific collaboration.

Participants from around the world representing Europe, Asia, North and South America, and Africa attended the 2023 meeting. The scientific program encompassed sessions on *Evolution, Information Processing Systems and Microbial Defence; Metabolism and Physiology; Biotechnology; Diversity and Ecology; Omics*; and *Thermophiles and Astrobiology*. Both established and early-career scientists contributed to the discussions, reflecting the diversity and breadth of research within the thermophile community.

The Congress provided an engaging forum for data presentation, conceptual exchange, discussion of research gaps, networking, and the formation of new collaborations. A friendly, inspiring, and inclusive atmosphere offered PhD students and early-career researchers an excellent opportunity to showcase their work through oral and poster presentations and to interact with leading experts from across the globe. Significant efforts were also made to incorporate sustainable development principles, with a strong emphasis on diversity and equality in speaker selection, session chairing, and the composition of the poster committee.

The *FEMS Microbiology Ecology* thematic issue on thermophiles was launched in conjunction with the conference to highlight key topics in thermophilic research through a collection of original research articles.

In this issue, Böer and colleagues (2024) reported on the isolation and characterization of novel strains of thermophilic, acetogenic *Moorella*, demonstrating their potential for biotechnological applications. Seven new *Moorella* strains were isolated from diverse environments, leading to the description of a new species, *M. carbonis*. The study also presented high-quality genome assemblies and identified potentially unique metabolic traits. Furthermore, physiological analyses revealed isolate-specific characteristics and, notably, documented prophage activity within the genus *Moorella* for the first time (Böer et al. 2024). Lassoued and co-authors (2024) focussed on exploring the diversity and interactions between cyanobacteria and heterotrophic bacteria in microbial mats of a hydrothermal pool at Ksar Ghilane, located in the Grand Erg Oriental, Tunisia. Although natural samples revealed a rich diversity of cyanobacterial species, laboratory cultivation resulted in the enrichment of selected genera, including *Leptolyngbya, Nodosilinea*, and *Arthronema*. The study highlighted the pivotal role of cyanobacteria within thermophilic microbiomes of hydrothermal pools, particularly through their metabolic exchanges with heterotrophic bacterial partners, predominantly from the phyla *Proteobacteria* and *Actinobacteria* (Lassoued et al. 2024).

The paper of Kristjansdottir et al. (2025) reported a genome-scale metabolic reconstruction of two strains of the thermophilic bacterium *Rhodothermus marinus* DSM 4252^T^ and the genetically amenable variant, ISCaR-493. The study provided detailed insights into sugar metabolism and carotenoid biosynthesis, among other metabolic pathways. Carotenoid production was found to vary significantly under different growth conditions; notably, supplementation of pyruvate to a glucose-based medium resulted in increased cell density. In addition, the presence of pyruvate, the use of alginate as a carbon source, light exposure, and nutrient starvation all contributed to enhanced carotenoid yields. Importantly, the previously unidentified *dxs* gene in the carotenoid biosynthetic pathway of *R. marinus* ISCaR-493 was successfully cloned from *Thermus thermophilus* using a shuttle vector, leading to further increases in carotenoid production (Kristjansdottir et al. 2025). Ciuchcinski et al. (2024) conducted a comparative analysis of plasmid identification tools applied to the study of microbial communities inhabiting deep-sea hydrothermal vents along the Arctic Mid-Atlantic Ridge. Plasmid sequences from 14 environmental samples were identified and characterized, enhancing our understanding of the role of the plasmidome in shaping microbial diversity, ecology, and adaptive mechanisms within extreme ecosystems (Ciuchcinski et al. 2024). Khusnutdinova et al. (2024) investigated the biochemical characteristics, resolved protein crystal structures and phylogenies, of glycosyl hydrolases (GH1) from the hyperacidophilic archaeon *Cuniculiplasma divulgatum* S5^T^, uncovering several significant insights. Phylogenetic analysis revealed that *Cuniculiplasma* GH1 enzymes cluster with homologues from thermophilic and hyperthermophilic archaea. Notably, two of the characterized enzymes exhibited optimal activity at 50°C, supporting the hypothesis that *Cuniculiplasma* may have evolved from high-temperature ancestors. These intracellular enzymes showed their highest activity toward cellobiose, suggesting a broader substrate range than previously recognized for *Cuniculiplasma*, which was once thought to rely solely on oligo- and polypeptides as carbon sources. Growth experiments further demonstrated that the addition and intracellular utilization of cellobiose increased biomass yield by ca. 50%. Collectively, these findings highlight a more substantial role of *Cuniculiplasma* in carbon cycling and emphasise its ecological importance in acidic environments, particularly through the uptake of cellobiose, a key intermediate in cellulose degradation (Khusnutdinova et al. 2024). Sunada et al. (2025) investigated sulfuric acidic hot springs in Japan and successfully isolated and identified several heterotrophic unicellular eukaryotic species belonging to diverse taxa. The study revealed independent evolutionary trajectories among the four isolated organisms, *Allovahlkampfia* sp., *Nuclearia* sp., *Parvularia* sp., and *Vannella* sp., each having acquired unique adaptations to elevated temperatures and low pH, enabling colonization of geothermal sulfuric environments. Additionally, other eukaryotic species, including *Platyophrya* sp. and *Neobodo*, were detected in these extreme habitats despite previously being characterized as mesophilic. An intriguing finding was that all species, except *Neobodo sp*., appeared to rely on *Cyanidiococcus* sp., the predominant member of microbial communities in these ecosystems (Sunada et al. 2025).

The study by Rosa et al. (2025) examined the survival of the thermophilic soil bacterium *Parageobacillus thermoglucosidasius* in the rhizosphere of tomato plants under fluctuating temperature conditions. Targeted metabolomic analyses demonstrated that plant exudates enhanced bacterial survival at 20°C–25°C by more than 23-fold, suggesting a potential mechanism for the persistence of thermophilic bacteria in heated soils during cooler periods (Rosa et al. 2025).

Taken together, the papers presented in this thematic issue encompass a broad spectrum of research on thermophilic and thermotolerant prokaryotic and eukaryotic organisms, addressing their diversity, ecology, evolution, and biotechnological potential. The studies employed an integrative range of methodologies, combining classical microbiological techniques such as enrichment, isolation, cultivation, and physiological assays with genomic, metagenomic, metabolomic, phylogenomic, and statistical analyses, as well as plasmid annotation, biochemical characterization of enzymes and resolving their crystal structures. These multifaceted approaches exemplify the modern, interdisciplinary nature of thermophile research. Collectively, the contributions in this issue highlight recent advances in the field and delineate key knowledge gaps that remain to be explored.
